# Revealing the Mechanical Properties of Emulsion Polymer Isocyanate Film in Humid Environments

**DOI:** 10.3390/polym10060652

**Published:** 2018-06-11

**Authors:** Jing Guo, Hongjiu Hu, Kefeng Zhang, Yaolong He, Xingming Guo

**Affiliations:** 1 Shanghai Institute of Applied Mathematics and Mechanics, Shanghai University, Shanghai 200072, China; m18800207545@163.com (J.G.); zhangkefengbjtu@163.com (K.Z.); yaolonghe@shu.edu.cn (Y.H.); xmguo@shu.edu.cn (X.G.); 2 Shanghai Key Laboratory of Mechanics in Energy Engineering, Shanghai 200072, China

**Keywords:** emulsion polymer isocyanate, tensile properties, post-cure treatment, relative humidity, viscoelastic constitutive equation

## Abstract

Knowledge of the mechanical behaviors of polymer film in humid environments is of great significance for predicting the long-term performance of emulsion polymer isocyanate (EPI) as a high-performance wood adhesive. A tri-copolymer latex was cross-linked by the general polymeric methylene diisocyanate (*p*-MDI) and aqueous emulsified isocyanate (EMDI) at different loadings for preparing EPI. Furthermore, a series of uniaxial tension tests under different relative humidity (RH) were carried out on cured EPI samples before and after post-curing treatment, and the corresponding chemical structure, as well as the microstructure of polymers, was investigated in detail. In addition, a constitutive equation was formulated to calculate the viscoelastic characteristics of the adhesive layer. The results indicate that the EPI films reveal various kinds of intrinsic deformation as RH increases, and the tensile rupture stress and stiffness would obviously decrease, even at cross-linker weight ratios of up to 20%. Furthermore, the moisture resistance could be markedly improved by increasing the isocyanate content and post-cure. Importantly, EMDI-cross-linked film not only exhibits much better mechanical properties than that containing *p*-MDI at 0–80% RH, but is also more sensitive to post-cure. Finally, the derived viscoelastic model could efficiently track moisture-dependent stress-strain curves of EPI films, and the obtained relaxation time further reveals the influence mechanism of isocyanate and post-cure on the mechanical response of the cured polymer under moist conditions.

## 1. Introduction

Emulsion polymer isocyanates (EPI) are composed of a water-based polymer emulsion and an isocyanate cross-linker. There is a remarkably strong interest in the application of EPI adhesives in solid wood processing and wood-based panel production for furniture manufacturing. The outstanding characteristics (high adhesion strength, good heat resistance, and environmental- soundness) make EPI one of the most promising choices for adhesives. It should also be noted that EPI-bonded wood products comprised of structural-composite lumber and cross-laminated timber are commonly used for buildings and bridges in Europe and North America [1]. As such, during their service life, the bond quality is influenced by the humid outdoor climate. Therefore, the moisture resistance of EPI has been extensively investigated in the past few decades.

To improve the bonding performance of EPI in different humid conditions, considerable efforts have been made to optimize the adhesive formulation. For example, Hu et al. investigated the warm and boiling water resistances of EPI adhesives for bonding rose wood, in which vinyl acetate homopolymer and copolymer emulsion were cross-linked by three types of polymeric methylene diisocyanate (*p*-MDI) respectively. It was found that aqueous emulsified isocyanate (EMDI) exhibits better cross-linking performance, followed by *p*-MDI mixed with organic solvent. The compression shear strength and percent wood failure are improved as the polyvinyl alcohol (PVOH) content increases, but excess PVOH impairs resistance to warm/boiling water [2]. The amount of isocyanates in the EPI will also notably affect bond performances. Krystiofiak et al. showed that the moisture resistance of the glue line increases with increasing loading of cross-linker [3]. The choice of emulsion will also influence the mechanical properties of EPI joints under both dry and wet states. With the addition of PVOH and nano-CaCO_3_ to a whey protein isolate solution, Guo et al. developed a novel EPI adhesive which has wet compression shear strength which surpass the JIS K6806 standards [4].

The preparation of wood surfaces is also of high importance regarding the moisture-related durability of EPI-glued timber products. When evaluating the water resistance of EPI for bonding yellow poplar wood acetylated to different weight percent gains, Vick and Rowell found that wet shear strength values significantly increase at higher levels of acetylation when compared to the untreated one, while wet wood failures decrease markedly as acetylation levels increase [5]. In another study, Knorz et al. carried out tensile shear and delamination tests for EPI bonded ash assemblies after exposure to moisture, in which the wood components were prepared using peripheral planing, sanding and face milling respectively. The different surfacing techniques led to markedly different bonding surfaces, notably with regard to cell damage and the extent of fibrillation. The face-milled surfaces provided the highest moisture resistance due to a more homogenous stress distribution in the bond line [6].

Compared to the great deal of attention given to the bonding performance of EPI adhesives [2,4,7,8,9,10], only a few studies exist in which mechanical response of the cured polymer films is analyzed. One is a pioneering investigation made by Taki et al. [11,12]. They reported the relationship between the mechanical properties of cured adhesives and bond strength of EPI with different cross-linking densities over a wide temperature range. In another experiment, Umemura et al. performed dynamic mechanical analysis (DMA) measurements on cured EPI films, and observed a very small loss in storage modulus from 20 to 70 °C [13]. DMA revealed that the post curing resulted in wide variations in the viscoelastic behaviors of the polymer. Although the residual isocyanate would have been reduced by post-cure treatments, a complete consumption of isocyanate could not be achieved [14]. That it is to say, the concentration of cross-links in cured adhesives and the corresponding joint durability may further evolve during the servicing environment. However, it is still not clear how environmental moisture impacts the mechanical stability of cured EPI film. Knowledge of the deformation and failure behavior of the adhesive thin film under humid conditions not only allows for a better understanding of the bonding strength of EPI, but is also the basis of adhesive design and application for outdoor timber structures. Up to now, no previous studies have paid close attention to this issue.

As an essential mechanism in the deformation of polymer, yielding behavior may control fracture toughness by its effects on the crazing and micro-crack propagation. It is important to understand, on a molecular scale, how the mechanical response of polymeric materials is dependent upon chemical structure, strain rate, and temperature. Therefore, considerable efforts have been made to study the connections between the yielding and nature of the polymer chain motions induced by temperature [15,16,17,18]. The similar effects of temperature and relative humidity on glassy stability [19,20,21] and durability regarding hydrothermal degradation [22] are well recognized. However, for various reasons, few studies in the literature exist on the molecular analysis of yielding behavior and the relaxation mechanism of a glassy polymer in a mechanical way which takes RH variation into account.

In this study, we focus on exploring the role of moisture on the mechanical response of EPI film, aiming to provide a simple and accurate basis for theoretical modeling and applications of EPI-bonded wood products. A tri-copolymer latex was cross-linked by the polyisocyanate at different types and loadings for preparing EPI. In addition, a series of uniaxial tension tests under different RH were performed to determine the mechanical properties of EPI cured samples before and after post-cure treatment, and the glass transition relative humidity (RH_g_), chemical structure, and microstructure of polymers were checked using DMA RH scans, Fourier transform infrared spectroscopy (FTIR) and optical microscopy respectively. Moreover, a constitutive equation was formulated to quantitatively describe the moisture-effected stress-strain curves of EPI films, and the obtained relaxation time was used to unveil the effect mechanism of cross-linker and post-cure on the mechanical properties of the polymer in humid environments.

## 2. Experiment

### 2.1. Materials

(1) Main component: vinyl acetate/butyl acrylate/hydroxypropyl methacrylate tri-copolymer latex (xPVAc, GCLOCK2000, Gclock Adhesive Company, Ganzhou, China), containing approximately 30% (wt.) of CaCO_3_, as well as 10% (wt.) of PVOH as a protective colloid and the main provider of hydroxyl groups. The aqueous vinyl latex was synthesized by Gclock Adhesive Company (Ganzhou, China) and was used as received. It had a solid content of 52.5%, viscosity of 7500 mPa·s (at 25 °C), and pH of 6.3.
(2) Cross-linkers: EMDI (Rubinate 9259) and *p*-MDI (Rubinate 5005), supplied by Huntsman Polyurethanes (China) Ltd. (Shanghai, China), were employed as received. The characteristics of both cross-linkers are shown in Table 1.

### 2.2. Preparation of Samples

EPI adhesives were prepared by adding the cross-linker to the main component at cross-linker weight ratios of 0%, 5%, 10%, 15%, and 20%, respectively. The two components, after metering, were mixed for 30 min by an electric agitator, and degassed for 30 s. The films were prepared on Teflon-coated glass plates and dried in a constant temperature and humidity chamber at 40 °C for 48 h to evaporate the water. Subsequently, some of the samples were placed in the chamber at 140 °C for 24 h to further remove residual isocyanate groups in EPI. Finally, all the cured films, with dimensions of 20 mm × 5 mm × 0.04 mm, were kept in a desiccator with freshly dried silica gel at ambient temperature as samples for testing. 

### 2.3. Test Methods

The mechanical measurements were carried out by a film/fiber tension clamp on a DMA (TA Q800, TA Instruments-Waters LLC, New Castle, DE, USA) with a DMA-RH Accessory, where the RH regulation of the sample chamber was realized by controlling pure N_2_ and a saturated water vapor mixture of different proportions. The humidity accuracy was ±3% at 0–90% RH and ±5% RH above 90% RH.

#### 2.3.1. Uniaxial Tension

The mechanical tests were conducted in different RHs at ambient temperature. The samples were first equilibrated at 0% RH for 60 min, and then the relative humidity in the testing chamber was adjusted to fixed values of 0%, 20%, 40%, 60%, and 80% RH. After 90 min equilibration, uniaxial tension was carried out at a strain rate of 2% under these various constant RHs.

#### 2.3.2. DMA RH Scans

DMA tests were carried out under strain mode at a frequency of 1 Hz and amplitude of 20 m. For the RH scans under a controlled temperature, specimens were quickly dried in a measuring chamber at 0% RH, 25 °C for about 60 min, after which RH scans were performed isothermally at the rate of 1.0% RH/min, with the humidity ranging from 0% RH to 90% RH.

#### 2.3.3. FTIR

FTIR spectra were measured with an Avatar Nicolet 380 spectrometer (Thermo Fisher Scientific, Waltham, MA, USA) using the KBr tablet method. Each sample was scanned 16 times with a resolution of 4 cm^−1^ between 400 and 4000 cm^−1^. The obtained spectra were normalized, with the peak height of the C–H stretching band at approximately 2922 cm^−1^.

#### 2.3.4. Optical Microscopy

The surface morphology of EPI films was observed on an optical microscope (Zeiss Smart zoom 5, Carl Zeiss, Jena, Germany), and the eyepiece used for image acquisition tasks was PlanApo D 5x/0.3 FWD 30 mm. These image acquisition tasks were carried out by graphic stitching under free examination modes. Mixed illumination was also used in the tests and the magnification 200×. The obtained optical microscopy images were then transformed into binary images using a MATLAB program based on the region growing method.

## 3. Results and Discussion

### 3.1. Effects of Types and Loadings of Polyisocyanate

The mechanical behaviors of the bonding layer are crucial to the comprehensive understanding of the complicated deformation response of EPI adhesive in a moist environment. Therefore, a series of uniaxial tension tests under different RHs (0%, 20%, 40%, 60% and 80%) were carried out on the EPI cured samples, which were cross-linked by EMDI or p-MDI with 0%, 5%, 10%, 15% and 20% of loadings, respectively. 

**Figure 1:**
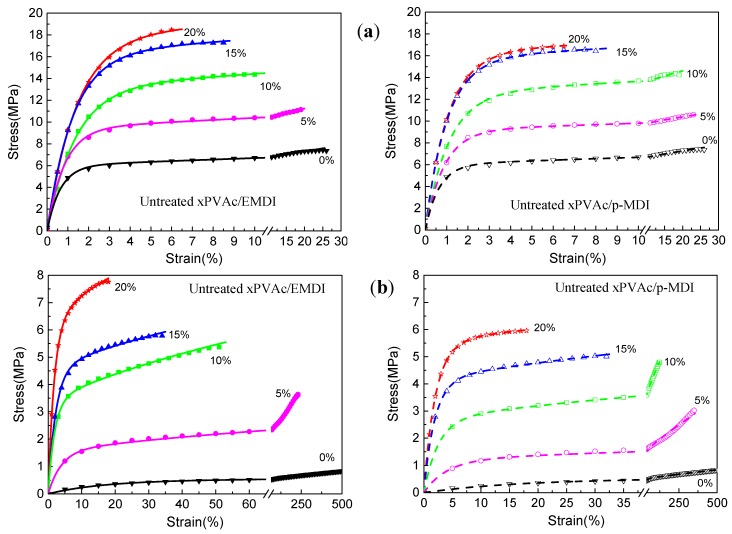
Effect of cross-linker on the tensile curves of cured EPI films at (**a**) 0% RH and (**b**) 80% RH (dot: experimental data, solid line: calculated by Equation (2)).

Figure 1a shows the mechanical behavior for dry EPI specimens. As the isocyanate content increases, the engineering stress-strain curves of polymer film have noticeably changed. For the low cross-linker loadings (0–5%), the raised strain causes the stress to quickly enlarge, followed by a relatively long period of minimal stress increase until rupture. With the addition of more isocyanate, the level of stress increases, and then plateau gradually shortens and disappears. This indicates that the cured films of aqueous vinyl emulsion are gradually transformed from a thermoplastic polymer to thermoset resin owing to the cross-link reaction between the hydroxyl groups (–OH) in polymer and isocyanate groups (–NCO). Moreover, compared to the standard version, the aqueous emulsifiable polymeric isocyanate exhibits better cross-linking effects, where the EPI films have relatively higher strength and stiffness.

**Figure 2:**
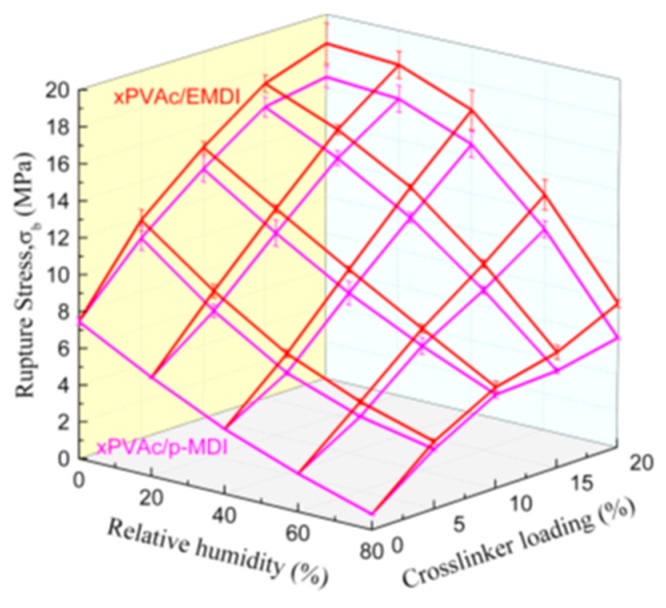
Effect of EMDI (red line) and *p*-MDI (magenta line) on the rupture stress of EPI film at different relative humidity.

Under high humidity conditions, due to the penetration of water molecules, scissioning of chain segments and the destruction of cross-links occur, resulting in the deterioration of the mechanical properties of the polymer. Thus, the tensile strength of the EPI films is greatly weakened, and the fracture strain increases considerably, as shown in Figure 1b. Moreover, it can be observed that the aqueous polymer would undergo various kinds of intrinsic deformation as the amount of isocyanate increases. Similar to the main component (xPVAc), the EPI film with 5% cross-linker displays the typical behavior of a soft elastomer, of which the fracture happens at the point of very high strain value. The values of $\varepsilon_{f}$ for xPVAc/EMDI and xPVAc/*p*-MDI are 240% and 350% at 80% RH, respectively, which are separately 12 times and 15 times of those when the compounds are in a dry state. However, within the range of higher cross-linker loading (15–20%), the polymers are neither rubber-like nor glassy, where the stress steeply goes up to the fracture point corresponding to a strain of 18–34%. It is much less than the value (240–500%) at low isocyanate addition levels (0–5%). At an intermediate cross-linker loading (10%), the specimen cross-linked by *p*-MDI bears a notably hyperelastic material with $\varepsilon_{f}$ of 125%, which is far larger than that of EMDI. The influences of cross-linker type and loading on the moisture resistance of cured film are further compared in Figure 2.

After EPI is applied on the substrate, as H_2_O is evaporated into the environment and reacts with the isocyanate groups as shown in Equation (1), the water content in the adhesive layer decreases, and tiny particles of polymer gradually form a film; while –OH cross-links with –NCO, the result forms the urethane bond [2]. These reactions and related chemical structures of the EPI films during curing the process are illustrated in Equations (2) and (3) respectively, by which not only xPVAc (vinyl acetate/butyl acrylate/hydroxypropyl methacrylate tri-copolymer latex), but also PVOH in the main component, can be cross-linked by the polyisocyanate to increase their molecular weights and establish the stable crosslinking networks after fabrication. As indicated in Figure 2, with the aid of polymeric isocyanate, the rupture stress ($\sigma_{b}$) remarkably improves the adhesive film under different relative humidity conditions. The magnitude of $\sigma_{b}$ at 0% RH is 46.4% higher in the EPI film with 5.0 wt % *p*-MDI compared to the neat xPVAc sample. As the weight ratio of cross-linker increases to 20%, the relative difference in $\sigma_{b}$ between EPI and its main component amplifies to 122.2%. Noticeably, with increasing EMDI or *p*-MDI content, the strength of dry film continuously increases. This is different to the effect of polymeric isocyanate on the bonding performance of xPVAc-based EPI at ambient temperature, which first rises then descends as the weight ratio of cross-linker increases from 0 to 20.0% [2]. This may be ascribed to the fact that more residual stress has arisen from the shrinkage of the adhesive layer due to the relatively higher curing rate as the EPI is coated on the surface of the dry wood specimen, where a great deal of water molecules are rapidly absorbed by the adherend. Meanwhile, not only noncovalent interactions, such as electrostatic interaction, hydrogen bonding, hydrophobic attraction, and van der Waals interaction [23], but also chemical crosslinking occur on the interfaces between wood and EPI film. However, if the evaporation rate of water in the EPI adhesive is too low, the coalescence of particles in the emulsion would occur after the chemical cross-link between the isocyanate group and active hydrogen in the main component. Consequently, the crosslinking reactions on the surface of the particles may impede the formation of a continuous film. Therefore, it is important in the design of the robust EPI bond-line to control the water-loss speed and the crosslinking reactions well with the diffusion and coalescence of tiny particles, film formation, and emulsion migration into the porosity of the surface of the adherend.

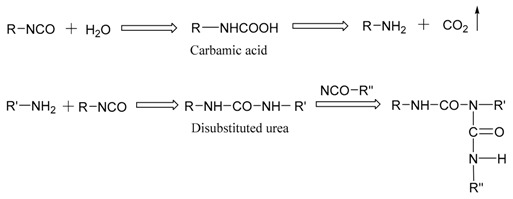
(1)

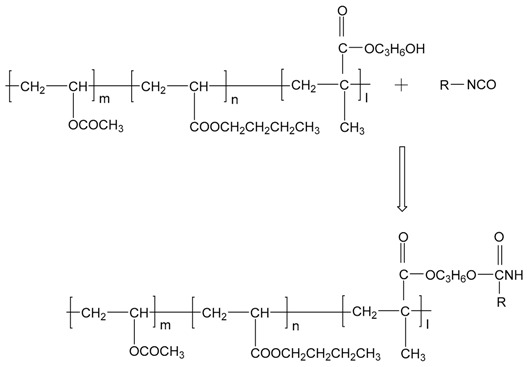
(2)

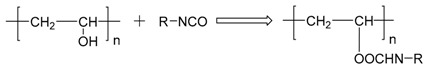
(3)

**Figure 3:**
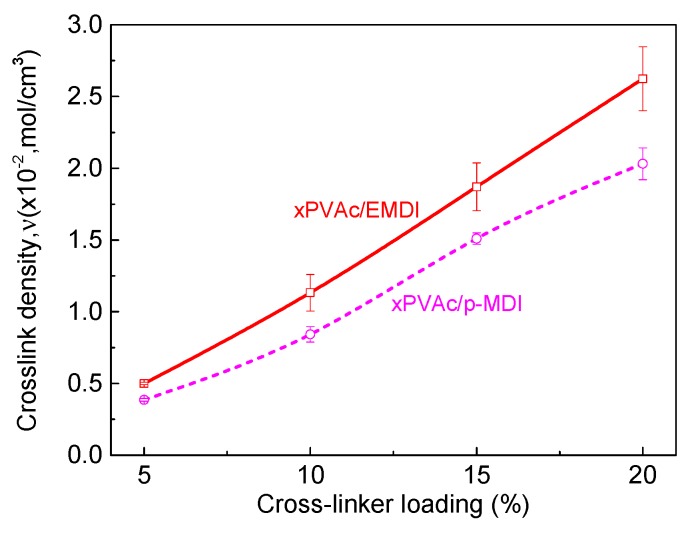
Crosslink density of EPI films at 80% RH with respect to crosslinker loading.

When water-based polymer film is subjected to moisture, because of the strong attraction of the water molecules to the amorphous chains of aqueous polymer, the electrostatic forces create a mutually exclusive system, forcing molecular chains to become a looser framework, and leaving extra free volume. Therefore, the small molecules can penetrate into the non-crystalline zone and reduce the local density of the system, which results in the strength of the cured film decreasing through an increase in the relative humidity at isothermal conditions (see Figure 2). As anticipated, polymeric isocyanate is helpful in alleviating plasticization. Furthermore, it was found that the moisture resistance of EPI film is strongly dependent upon the cross-linker content. That is, the more isocyanate that is added, the higher the rupture stress of the cured film in humid conditions. To explore the effect of the cross-linker content on the moisture resistance of the EPI films, the cross-link density was used to describe the degree of cross-linking between the isocyanate groups and functional groups in xPVAc. The cross-link density refers to the number of cross-linked bonds per unit volume, and can be calculated using the Young’s modulus from the stress-strain test [24]. The evolution of the crosslink density as a function of the isocyanate content is shown in Figure 3.

As seen in Figure 3, as the isocyanate content increases, the crosslink density rises at an almost uniform rate. Importantly, the cross-linked structure is a very powerful barrier to the penetration of water molecules: the greater the crosslink density, the greater the difficult of penetration of water molecules. A highly cross-linked structure can also cause massive restrictions on chain movement, which means that the relative content of amorphous chains in the EPI samples is greatly reduced, and that the free volume among the macromolecular chains is also decreased. Because fewer water molecules can penetrate into samples with a higher cross-linker content, their strength in a humid environment can be maintained at a higher value.

Figure 2 shows that xPVAc/EMDI films have much better moisture resistance than xPVAc/*p*-MDI films with the same isocyanate, and that the difference tends to become more obvious as the cross-linker amount increases. This result may be interpreted as follows: On the one hand, as shown in Figure 3, the crosslink density of xPVAc/EMDI films is greater than that of xPVAc/*p*-MDI films throughout the cross-linker content range tested. Moreover, as the cross-linker addition increases, the gap in cross-link density between the two types of films gradually widens. Therefore, the water resistance of xPVAc/EMDI films is better. On the other hand, as the more non-polar cross-linker, *p*-MDI in the form of small particles is not easy to disperse into the polar aqueous main polymer component, and this situation is further exacerbated at higher isocyanate ratios. In EMDI, where the polymeric isocyanate is modified with non-ionic surfactants, the interface between the hydrophilic and lyophilic groups in the EPI adhesive is optimized. Thus, EMDI will facilitate the dispersion of the isocyanate into xPVAc and PVOH, and thus, enhance the contact probability and cross-linking reaction between the –OH of the aqueous polymer and –NCO in the cross-linker. Consequently, the mechanical properties of the cured film are improved by the higher crosslink density through its effect on the molecular mobility and the glass transition relative humidity.

**Figure 4:**
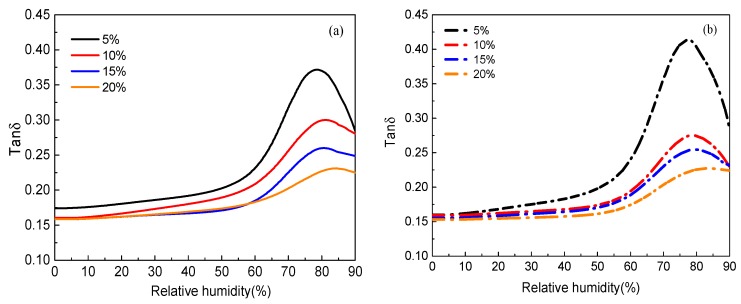
Effect of crosslinker content on RH-dependent loss tangent (tan*δ*) of (**a**) xPVAc/EMDI and (**b**) xPVAc/*p*-MDI.

Analogous to the glass transition induced by temperature change, external relative humidity variation and the resulting change in water content can also lead to a remarked viscoelastic state change of the EPI film, as seen in Figure 4. With the loading of cross-linker varying from 5 to 20%, the introduction of isocyanate group crosslinks increased the width of the RH-dependent tan*δ*, and decreased the peak height, suggesting that the molecular weight increases and the molecular weight distribution broadens. It can be clearly observed that there exists the critical relative humidity denoted as RH_g_, corresponding to the peak of loss tangent. Admittedly, the addition of polyisocyanate was helpful in increasing the glass transition relative humidity, causing a greater constraint on chain movement and a higher crosslink degree. Accordingly, the more isocyanate added, the higher the RH_g_. As anticipated, the RH_g_ value of xPVAc/EMDI is higher than that of xPVAc/*p*-MDI at the same cross-linker content.

**Figure 5:**
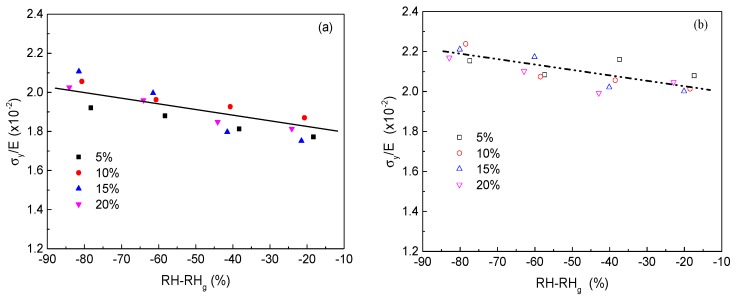
Evolution of yield stress normalized by the elastic modulus (σ*_y_*/*E*) versus RH-RH_g_ for cured film at different cross-linker content (**a**) xPVAc/EMDI and (**b**) xPVAc/p-MDI.

As stated above, experimental results show that an increase in relative humidity would lead to not only a decrease of mechanical properties, such as yield and rupture stress, but also of the glass transition of adhesive film. One may wonder whether there exists some connection between plastic behavior and the segmental mobility process. Inspired by the work of the Halary group [15,16,17,18], we have plotted the yield stress normalized by elastic modulus ($\sigma_{y}/E$) of cured film versus departure from main mechanical relaxation relative humidity ($RH-RH_{g}$) in Figure 5 for xPVAc/EMDI and xPVAc/*p*-MDI. The value of $\sigma_{y}$ was defined as the point where the stress passed at the obvious ‘knee’ in the stress–strain curve (because a maximum in stress was not observed in Figure 1). It can be seen that almost all the plots of $\sigma_{y}/E$ against $RH-RH_{g}$ lie on a unique master curve for xPVAc/EMDI film with different isocyanate loadings; xPVAc/*p*-MDI shows a similar trend. All the EPI cured films with various cross-link densities exhibit almost the same profile of evolution $\sigma_{y}/E$ as a function of ($RH-RH_{g}$), which implies there is a correlation between the yielding behavior and segmental mobility in the β-relaxation processes induced by moisture.

### 3.2. Effect of Post-Cure Treatment

Although the NCO groups can be so reactive that they react with almost all polar substances in the wood and aqueous polymers such as water and hydroxyl groups, the isocyanate absorption band at 2270 cm^−1^ was still detected in the spectrum of an EPI film stored for 60 days at ambient temperature [14]. As a result, the residual NCO groups may further react with environmental moisture, leading to the presence of minor defects in the adhesive layer due to carbon dioxide (CO_2_) gas arising from this reaction, as shown in Equation (1). Therefore, to improve the durability performance of EPI-bonded structures under operating conditions, reducing the amount of unreacted isocyanate in the glue-line is essential. Importantly, the post-curing treatment, i.e., by heating at 120–180 °C, could decrease the content of residual NCO groups in an EPI film sample, thereby contributing to the higher dynamic storage modulus [14]. How the moisture resistance of EPI films with different cross-linker types and loadings would be affected, however, if this additional curing procedure is added, remains unclear. To answer this question, we compared the moisture-dependent rupture stress of xPVAc-based EPI films with and without the thermal treatment, and explored the evolution of the porosity on the film surface, the cross-link density, and residual isocyanate groups in the cured samples with different cross-linkers.

**Figure 6:**
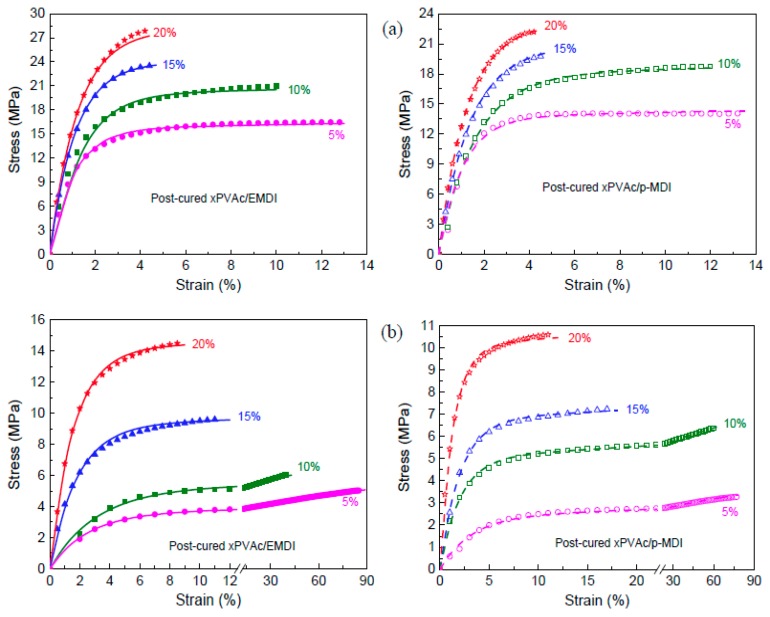
Representative stress-strain curves of post-cured EPI film at (**a**) 0% RH and (**b**) 80% RH (dot: experimental data, solid line: calculated by Equation (2)).

**Figure 7:**
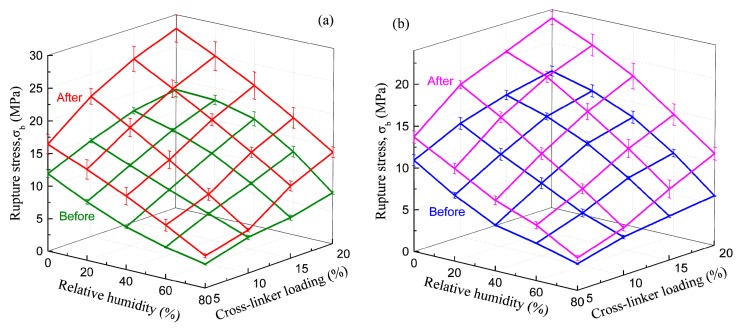
Effect of post-cure treatment on moisture-dependent rupture stress of (**a**) xPVAc/EMDI and (**b**) xPVAc/*p*-MDI.

To decrease the content of residual NCO groups in polymer film, EPI specimens were heated in an oven at 140 °C for 24 h, and then subjected to the same uniaxial tensile test. The representative engineering stress-strain curves are shown in Figure 6. Furthermore, to compare the effect of post-cure on the moisture resistance of EPI specimens with different cross-linker, the rupture stresses of xPVAc/EMDI and xPVAc/p-MDI films before and after the treatment were determined, as shown in Figure 7a,b, respectively.

As shown in Figure 7, in either a dry atmosphere or a humid environment, the rupture stress ($\sigma_{b}$) of EPI films cross-linked by both EMDI and p-MDI after post-cure is significantly higher than that of untreated specimens, which indicates that the treatment can generally enhance the mechanical properties of EPI films. After heating, $\sigma_{b}$ of dry film with 5% EMD increases 38%. As the weight ratio of EMDI to the main component increases to 20%, it would boost by approximately 51%, which is considerably greater than that of the 5% EMD-cross-linked specimen. The EPI films containing *p*-MDI also show the similar trend, but relatively less improvement. This observation indicates that the treatment effectively promotes the cross-linking reaction between the –OH and the functional groups in the main component, and may obtain additional intramolecular crosslinking, as indicated in Equation (4). As stated by Wei and Haag [23], the intra-polymer interactions such as physical and chemical crosslinking can also substantially improve the stability of the coating. It is worth noting that all of the treated specimens in a dry environment show a monotonic strengthening as cross-linker loading increases, indicating that the post-cure would not result in over-crosslinking of EPI specimens, even with 20% polymeric isocyanate.



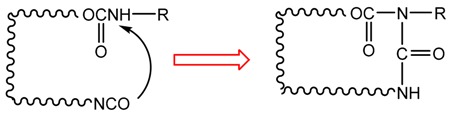
(4)


Due to plasticization effect of moisture, the rupture stress of the post-cured films also decreases as the relative humidity increases. As anticipated, heating treatment can significantly increase moisture resistance of EPI specimens. Certainly, the degree of improvement depends upon the water absorption in the polymer films. At 80% RH, the treated specimens with 5–10% cross-linker show increases by 20–30% in $\sigma_{b}$. However, with the aid of a higher cross-linker content (20%), there is a dramatic increase. The wet strength value of post-cured xPVAc/EMDI and xPVAc/*p*-MDI are increased by approximately 80% and 60% respectively, compared to their untreated specimens. It was also found that the treatment is more effective for the film cross-linked by EMDI than p-MDI at other loadings (5%, 10% and 15%). 

To determine the effect mechanism of the post-cure on the mechanical behavior of EPI films, it is necessary to systematically analyze the changes in the structure and composition of the polymer. As the cross-linked structure is an effective barrier to water molecule infiltration, the higher the crosslink density, the more difficult the diffusion by water molecules. The first aim of the following discussion was the clarification of the changes in the cross-link density (υ) of xPVAc cross-linked by EMDI or *p*-MDI, before and after post-curing treatment at various isocyanate loadings.

**Figure 8:**
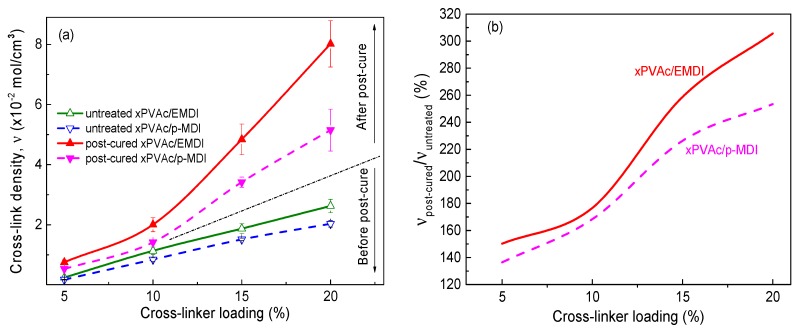
Effect on the (**a**) cross-link density and (**b**) ratio cross-link density of EPI film before and after post-curing at 80% RH.

Figure 8 illustrates the effect of post-curing on the crosslink density (υ) of two kinds of EPI specimen at 80% RH. Upon 24 h of heating at 140 °C, the value of υ increases substantially as a consequence of post-curing and the formation of new crosslinking structures, and cured effect sharply enlarges as more cross-linker is added. Importantly, the degree of cross-link of post-cured film with 15% cross-linker is noticeably higher than that of the untreated one with 20% cross-linker. The result may be very helpful in developing cost-effective EPI-bonded wood products, especially as the price of polyisocyanate has increased to approximately USD 5000 per ton at present.

**Figure 9:**
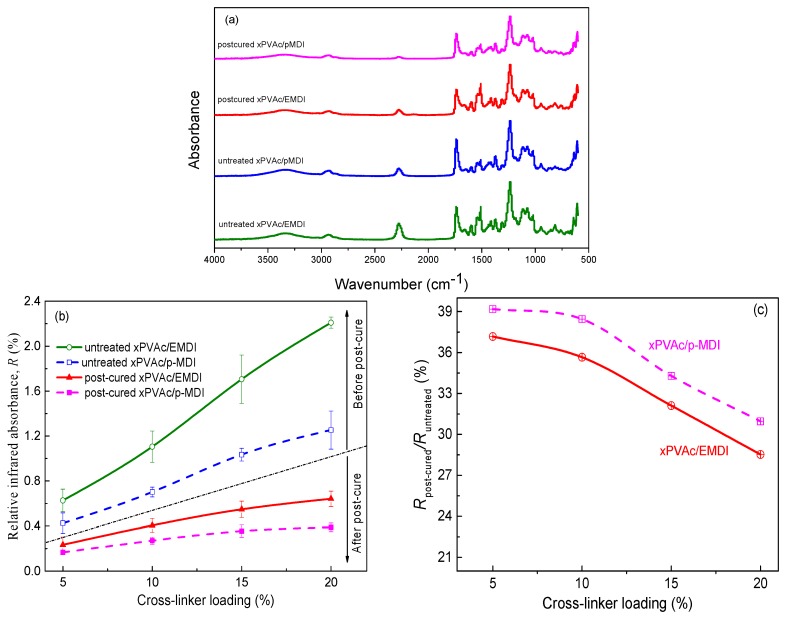
(**a**) FTIR spectra of; (**b**) relative infrared absorbance (R) and (**c**) its ratio for NCO in EPI films after and before post-cure.

Good dispersion of EMDI in vinyl acetate-based tri-copolymer latex facilitates not only the chemical reaction of –NCO with active hydrogen in EPI in a humidity environment, but also potential intra-crosslinking of the polymer film, subsequently expediting the curing process and an increase in υ. As seen in Figure 8, it is reasonable to conclude that xPVAc/EMDI film subjected to moisture results in a higher crosslink degree, as compared with xPVAc/*p*-MDI at the same isocyanate content. After being thermally treated for a day, it is observed that the ratio of the cross-link density ($\nu_{post-cured}/\nu_{untreated}$) of xPVAc/EMDI is also larger than that of xPVAc/*p*-MDI, and the difference enlarges as the cross-linker loading increases. The reason for this is that within the post-curing period, a much stronger cross-link reaction take places in the EPI film with EMDI, indicating that there may be more residual isocyanate in the untreated polymer. For the purpose of probing the difference in –NCO residue of the EPI films, the chemical structure was checked using FTIR. The peak value of CaCO_3_ at 875 cm^−1^ [25] was used as the benchmark for spectrum normalization, and the normalized curves are represented in Figure 9a. Seemingly, the reaction from the isocyanate can be perceived due to the presence of absorption bands in the C=O stretching in the range of 1740–1620 cm^−1^ and N-H stretching around 3300 cm^−1^ [14]. However, these characteristic bands could not be distinguished because the C=O stretching region was covered with a huge band due to CaCO_3_ being centered at 1400 cm^−1^, and the N-H stretching bands overlapped with the broad band assignable to water [26]. The following therefore focuses on the evolution of the relative intensity of the peak at 2270 cm^−1^ assigned to an asymmetric isocyanate stretching; the relative absorption ratio of the peaks, $R=A\left(2270cm^{-1}\right)/A\left(875cm^{-1}\right)$, interpreted as the amount of residual NCO groups in cured EPI film before and after post-cured treatment, was plotted as shown in Figure 9b.

**Figure 10:**
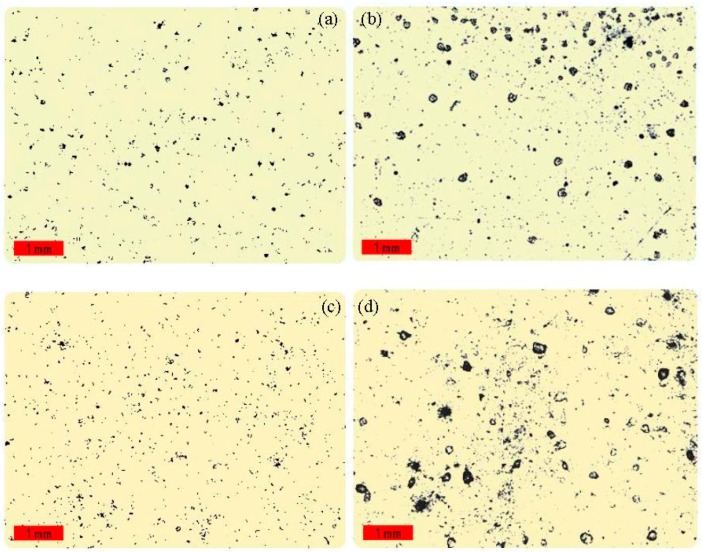
Optical photographs of (**a**) untreated xPVAc/EMDI; (**b**) untreated xPVAc/*p*-MDI; (**c**) post-cured xPVAc/EMDI and (**d**) post-cured xPVAc/*p*-MDI (black dots: micro-pores).

From Figure 9b, under the same cross-linker loading, xPVAc/p-MDI films reveal markedly lower relative infrared absorbance (R) than xPVAc/EMDI before post-curing, indicating that the –NCO depleted much faster for the *p*-MDI based EPI film during the preparing process. As its cross-link density is evidently smaller compared to that of the film with EMDI, it may be deduced that more of the cross-linker of xPVAc/*p*-MDI reacted with H_2_O in this stage. The inference can be further verified by the higher porosity in this type of specimen (see Figure 10). After post-cure, the magnitude of R drops sharply, while the ratio of relative infrared absorbance ($R_{post-cured}/R_{untreated}$) for –NCO in the xPVAc/EMDI and xPVAc/*p*-MDI films after and before thermal treatment at 24 h is about 37% and 39%, respectively, and it gradually decreases as the cross-linker loading increases. Therefore, post-curing treatment is necessary for EPI, especially with high isocyanate content. Furthermore, the treated xPVAc/EMDI still has relatively higher residual isocyanate. However, it can be seen from Figure 9c that the value of $R_{post-cured}/R_{untreated}$ is lower than the EPI film with *p*-MDI, demonstrating that more isocyanate reacted in post-cured xPVAc/EMDI films than in xPVAc/*p*-MDI. This may be attributed to the fact the water vapor from the environment can also expediently penetrate into the relaxed EPI with EMDI in treatment at 140 °C, resulting in there being almost no difference in the reactivity of water molecules with the two kinds of polyisocyanate. Meanwhile, due to the uniformly distributed particles and higher concentration of residual isocyanate, more –NCO in xPVAc/EMDI was consumed by the functional group in the polymer than in xPVAc/*p*-MDI. Hence, the post-curing effect on EMDI-crosslinked films is stronger than on that containing *p*-MDI, and the treated film is more densely cross-linked, contributing to a more obvious increase in moisture resistance.

As the post-cured cross-linking of EPI film is accompanied by the reaction between isocyanate and water as described in Equation (1), the micropore magnitude on the specimen surface will be considerably changed due to the released carbon dioxide gas. Therefore, the change in micropore distribution may reflect the extent of this reaction. Based on this hypothesis, we used a 3D optical microscope to observe the morphologies of the micropores on the surface of the EPI specimens with different cross-linkers before and after heat treatment, as shown in Figure 10. Distinct micropore distribution and morphology were observed on the film surface. Before post-curing, the micropores are very small and sparsely distributed, and the specimens with EMDI show smaller micropores than *p*-MDI, indicating that less isocyanate was consumed by water molecules during EPI film preparation. After treatment, some larger pores appear, and the number of micropores increases rapidly due to the reaction of residual NCO and moisture of the surroundings; there is also a comparative increase of micropores on the film surface for both types of EPI. This observation may confirm that post-cure facilitates residual isocyanate in xPVAc/EMDI preferentially reacting with the available group in the polymer, resulting in a higher degree of chemical cross-linking.

### 3.3. Model Analysis on the Moisture-Dependent Mechanical Response

In the previous section, EPI films with different cross-linkers were systematically subjected to quasi-static tensile tests in a variety of humid environments, and the corresponding stress-strain curves were obtained. However, the experiments could not encompass all relative humidity, making it necessary to establish a theoretical model that can predict the mechanical behavior of adhesive films. This section offers a constitutive model to quantitatively describe the moisture-dependent stress-strain curves of cured films; calculated relaxation time is used to determine the effect mechanism of cross-linker and post-curing on the water resistance of EPI.

**Figure 11:**
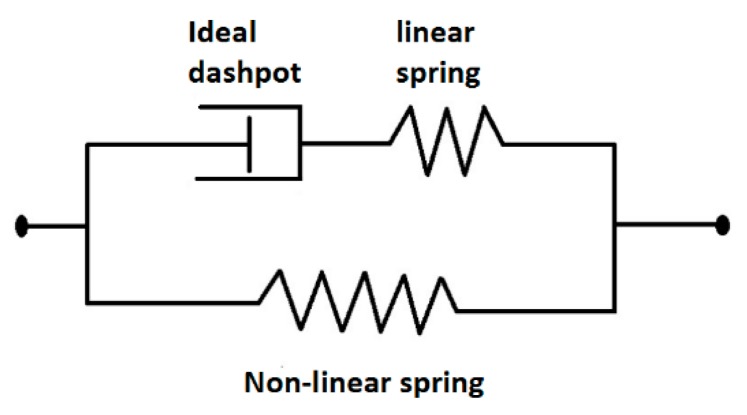
Rheological model for EPI film composed of two Maxwell elements in parallel.

As shown in Figure 1, the moisture-absorbed EPI samples display typical behavior of thermoplastic materials. Therefore, it is feasible to derive a viscoelastic constitutive equation based on the rheological model composed of two Maxwell elements in parallel (see Figure 11), and we can obtain the relationship between stress (σ) and strain (ε) in the equation below (2).

(5) $$\sigma =2c_{10}\left[(1+\varepsilon )-\frac{1}{{\left(1+\varepsilon \right)}^{2}}\right]+\dot{\varepsilon }\eta_{1}(1-e^{-\frac{\varepsilon }{m\frac{\eta_{1}}{E_{1}}}})$$

where $c_{10}$ is the stiffness of a non-linear spring, $E_{1}$ is the stiffness of a linear spring, $\eta_{1}$ is the viscosity of an ideal dashpot, and $\dot{\varepsilon }$ is the strain rate.

Equation (5) was used to describe all the uniaxial tensile test curves of the dry and wet specimen cross-linked by EMDI or *p*-MDI. The representative results are shown in Figure 1 and Figure 6, where the calculated values (solid lines) were in agreement with the experimental data (dots). The correlation coefficient close to 1 indicated that the constitutive model was accurate for calculating the mechanical behavior of EPI films in humid surroundings. As elaborated in the published works [27,28,29,30], relaxation time is an intrinsic property describing the hysteresis of chain segments of polymer or its composites under external stress. In order to investigate the moisture influence on the mechanical behaviors of EPI film in greater depth, we attempted to determine the relaxation time of a cured sample at 0–80% RH as follows.

The relaxation time (τ) of a viscoelastic solid is defined as the ratio of the system viscosity to the system stiffness. Therefore, we can use the material parameters $E_{1}$, $\eta_{1}$ and $c_{10}$ obtained by the fitted experimental stress-strain curves to determine the τ value of the EPI films in various conditions according to the Equation (6):

(6) $$\tau =\frac{\eta_{1}(E_{1}+c_{10})}{E_{1}c_{10}}$$

**Figure 12:**
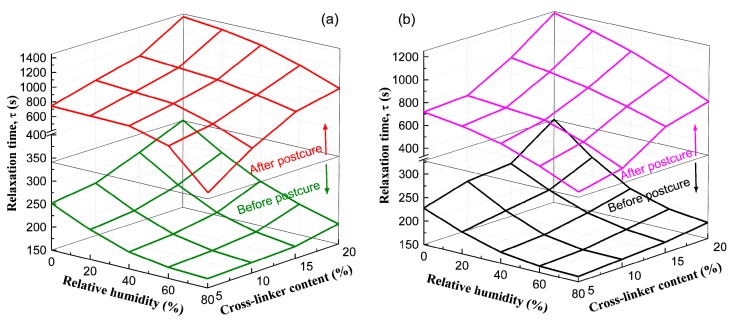
Changes in relaxation time against different RH and crosslinker content for (**a**) xPVAc/EMDI and (**b**) xPVAc/p-MDI films before and after post-curing treatment.

The change in relaxation time (*τ*) against RH for EPI film with different crosslinkers before and after the post-curing treatment is shown in Figure 12. The water molecules penetrating into EPI films form hydrogen bonds with the hydroxyl groups in the main component [31], by which the interchain and intra-chain bonding among amorphous chains in the polymer may be broken [32], and the τ magnitude descends by about 30–50% as RH increases from 0 to 80%. Furthermore, it was found that the relaxation time of EPI film lengthens with the increased polyisocyanate content, also verifying the effective cross-linking between NCO groups and active hydrogen in EPI. Adding more cross-linker or post-curing would cause a stronger interaction force due to the remarkable substantial increase in the number of cross-link bonds among molecular chains, meaning that the film would shrink further and the space around the network chains would be compressed. Under such a steric hindrance effect, the conformational transition of molecular chains is inhibited, and the motion of cross-links is more strictly constrained [33]. As anticipated, xPVAc/EMDI exhibits a slower relaxation process than xPVAc/*p*-MDI of the same cross-linker ratio, owing to the higher crosslinking capacity of emulsified polyisocyanate. Meanwhile, the relaxation times of all EPI specimens can be also greatly increased by post-curing.

**Figure 13:**
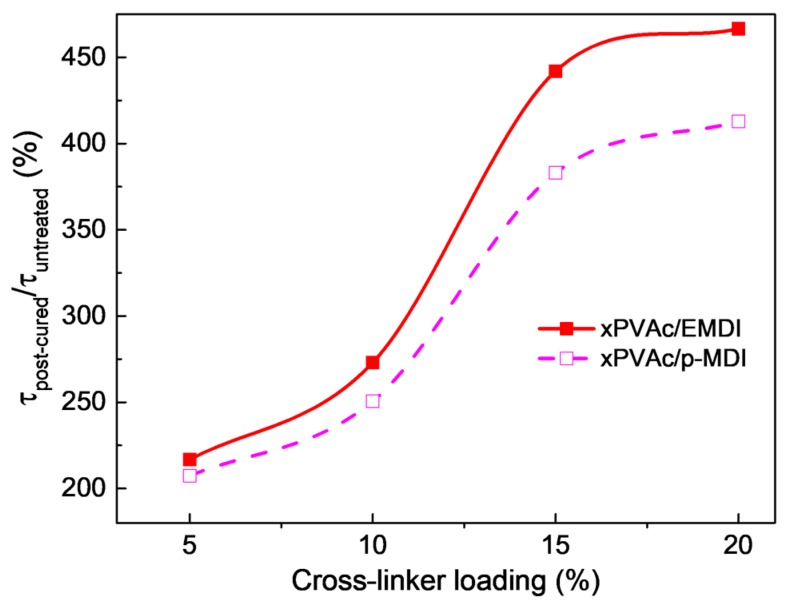
Effect of cross-linker loading on ratio of relaxation time for post-cured EPI film after and before post-cure.

In order to further compare the responses of different EPI specimens to post-cured treatment, we calculated the relaxation time and cross-link density of a post-cured film divided by those of an untreated one, as shown in Figure 13. With the increased loading of polyisocyanate, both the ratio of relaxation time ($\tau_{post-cured}/\tau_{untreated}$) and the ratio of crosslink density ($\nu_{post-cured}/\nu_{untreated}$), as shown in Figure 8b, of the polymer films is noticeably enlarged. That is to say, the higher the cross-linker loading, the better the treated effects. Moreover, the relative increment of both material properties for xPVAc/EMDI is higher compared to xPVAc/*p*-MDI at the same cross-linker loading, also validating the fact that the EMDI-crosslinked films is more sensitive to the post-curing process in terms of chain segments relaxation. Notably, due to coupling the variation of stiffness and viscosity of cured polymer film, the value of $\tau_{post-cured}/\tau_{untreated}$ is significantly larger than $\nu_{post-cured}/\nu_{untreated}$ for the identical EPI. Therefore, the relaxation time may be the best intrinsic parameter for characterizing the influence of isocyanate, of post-curing treatment, and of environmental conditions on the EPI polymer.

## 4. Conclusions

(1) With increasing relative humidity, typical plasticization still occurs for EPI with high isocyanate content, leading to a clear decrease in the tensile rupture stress and Young’s modulus of cured adhesive layer at ambient temperature.
(2) The moisture resistance of EPI film can be significantly enhanced not only by increasing the isocyanate content, but also by the post-curing treatment. Although xPVAc/EMDI has a higher amount of residual NCO groups in the cured film than xPVAc/*p*-MDI, it exhibits much better mechanical properties and cross-linking for the whole RH range, because aqueous emulsified isocyanate has led to a more efficient chemical reaction with the functional groups available in/on the emulsion polymer compared to the general polyisocyanate cross-linker.
(3) The introduction of isocyanate groups contributes to an increase in the glass transition relative humidity of EPI film, and the RH_g_ value of xPVAc/EMDI is higher than that of xPVAc/*p*-MDI at the same crosslinker loading due to a greater constraint on chain movement. Moreover, at a given $RH-RH_{g}$, the ratio of yield stress to elastic modulus does not depend markedly on the crosslinker type and loading. This also supports the idea that mobility and cohesion effects mainly govern yield behavior of polymeric materials from another viewpoint.
(4) Post-cure is found to efficiently reduce the residual NCO groups in cured film, resulting in a significant increase in the crosslink density and also the mechanical behavior of EPI. Notably, the post-curing effect on EMDI cross-linked films is stronger than on those containing *p*-MDI.
(5) The derived viscoelastic constitutive equation has quantitatively characterized the tensile stress-strain curves of EPI films under variable relative humidity conditions, and the related relaxation time can clarify the influence mechanism of the isocyanate type and loading, as well as post-curing treatment on the moisture resistance of the cured polymer.

These results may shed light on the complicated mechanical behaviors of emulsion polymer isocyanate film in humid environments, which is of great significance for predicting the long-term performance of EPI-bonded structures. This study also provides insights into the role of EMDI and post-curing treatment, facilitating proper engineering designs and applications for high-performance adhesives.

## Figures and Tables

**Table 1:** Characteristics of polymer isocyanates.

Cross-Linker	Solid Content, %	NCO, %	Functionality	Modification Method
Rubinate 9259	100	30.6	2.7	Aqueous emulsifiable
Rubinate 5005	100	30.5–32.5	2.6–2.7	Standard industrial
